# IL-33/ST2-mediated inflammation in macrophages is directly abrogated by IL-10 during rheumatoid arthritis

**DOI:** 10.18632/oncotarget.16299

**Published:** 2017-03-16

**Authors:** Si Chen, Bingni Chen, Zhongyang Wen, Zhong Huang, Liang Ye

**Affiliations:** ^1^ Institute of Biological Therapy, Shenzhen University, Shenzhen, China; ^2^ Department of Pathogen Biology and Immunology, Shenzhen University School of Medicine, Shenzhen, China; ^3^ Shenzhen City Shenzhen University Immunodiagnostic Technology Platform, Shenzhen, China

**Keywords:** IL-33, IL-33 receptor, IL-10, macrophage, rheumatoid arthritis, Immunology and Microbiology Section, Immune response, Immunity

## Abstract

IL-10 is an immunosuppressive cytokine produced and sensed by many immune cells and exerts a protective role in autoimmune diseases. However, the underlying mechanism by which IL-10 contributes to prevent the arthritic inflammation in macrophages is poorly understood. Herein we report on a novel anti-arthritic property of IL-10 through the inhibition of IL-33 signaling by macrophages during collagen-induced arthritis (CIA) development. We show that IL-33 expression rather than its receptor (ST2) is positively correlated with IL-10 level in active RA. IL-10 deficiency in mice leads to significant upregulation of IL-33 expression and aggravates the progression of CIA, while exogenous IL-10 treatment effectively diminishes IL-33 production in IL-10 knockout (IL-10^−/−^) CIA mice. We demonstrate further that the inhibitory effect of IL-10 in suppressing IL-33 production requires STAT3 activation in macrophages. Furthermore, IL-33 stimulated proinflammatory genes are notably increased in IL-10^−/−^ CIA mice, whereas macrophages treated with recombinant IL-10 exhibit decreased IL-33 amplified inflammation and inhibited IL-33 activated NF-κB signaling pathway. Our findings indicate that IL-10 act as a negative regulator of IL-33/ST2 signaling pathways *in vivo*, suggesting a potential therapeutic role of IL-10 in autoimmune diseases.

## INTRODUCTION

Rheumatoid arthritis (RA) is an autoimmune disease that is characterized by inflammatory cells infiltration of the joint, leading to cartilage and bone destruction [1]. Dysregulated macrophage responses have been shown to contribute to many autoimmune pathogenesis by the production of proinflammatory cytokines such as IL-33, ST2 and IL-1β, as well as proinflammatory chemokine MCP-1 production [2, 3, 4] Remarkably, depletion of macrophages alleviates the symptoms and severity of collagen-induced arthritis (CIA) in mice [5]. In fact, the frequency of macrophages is markedly elevated in the inflamed synovial tissues of patients with RA and positively correlated with disease pathogenesis and progression [6].

IL-33 is a new member of the IL-1 family and is recognized as a ligand for the ST2 receptor [7]. IL-33 and ST2 are broadly expressed in many immune cells, including macrophages, dendritic cells, mast cells, and Th2 cells [7]. Activated ST2 signaling by extracellular IL-33 may result in activation of NF-κB and MAPK (P38, ERK and JNK) in macrophages leading to a potential induction of pro-inflammatory cytokines (IL-1β) and chemokines (MCP-1) [7]. The IL-33/ST2 axis is involved in the pathogenesis of autoimmune diseases. Recent reports have found increased IL-33 and ST2 production in the serum and synovial tissue of patients with RA [8, 9]. The expression levels of IL-33 and ST2 seem to correlate with RA disease activity [8]. Administration of IL-33 induces the production of pro-inflammatory cytokines (IL-1β, MCP-1 and IL-6) and exacerbates the development of CIA in mice [9]. These data indicate that abrogation of IL-33/ST2 amplified inflammatory response represents a therapeutic target for RA.

IL-10 is a pleiotropic anti-inflammatory cytokine that produced by most hematopoietic cells, including macrophages, regulatory T and B cells, and mast cells [10]. The suppressible effects of IL-10 mainly depend on IL-10 receptor (IL-10R) to employing a STAT3-dependent manner [11]. Genetic mutation in IL-10 is linked to autoimmune diseases in human and mice that loss of IL-10 develops severe RA inflammation and augments disease progression [11, 12]. Although the roles of IL-10 in T and B cell function have been extensively studied, there is limited knowledge on the molecular basis of IL-10 on macrophages. Our previous studies demonstrated that IL-10 inhibits inflammatory factor synthesis in macrophages and restrains macrophages polarization toward the pro-inflammatory M1 phenotype [12, 13]. It is also possible that IL-10 govern inflammatory responses by blocking IL-33/ST2 axis in macrophages during RA.

In this study, we determine the direct contribution and mechanism of action of IL-10 in autoimmune inflammation by using IL-10 deficient mice. We describe for the first time IL-33 expression is positively correlated with IL-10 level in patients with active RA. We show an unanticipated role of IL-10 in the downregulation of IL-33 expression in macrophages. IL-10 suppressed expression of IL-33 requires STAT3 activation. Importantly, we found that macrophages are responsive to IL-10-STAT3 pathway for controlling IL-33 production. Furthermore, IL-33 induced the production of proinflammatory cytokines in macrophages is also blocked by IL-10.

## RESULTS

### IL-33 expression is correlated with the level of IL-10 in active RA patients

**Figure 1:**
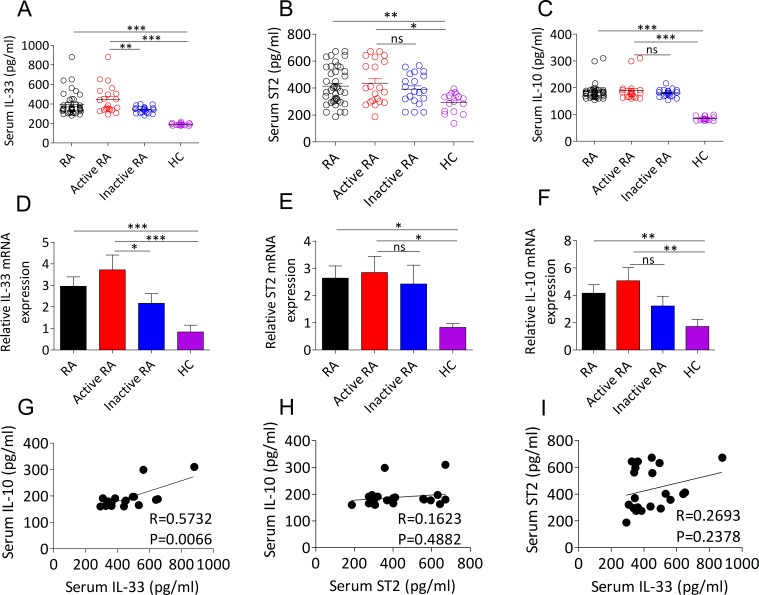
Expression levels of IL-33, ST2 and IL-10 in the serum and PBMCs from RA patients **A**., **B**., **C**. Serum IL-33 A., ST2 B. and IL-10 C. levels from RA patients (*n* = 41) and HCs (*n* = 16) were quantified by ELISA. Symbols represent individual samples. **D**., **E**., **F**. The mRNA expressions of IL-33 D., ST2 E. and IL-10 F. in PBMCs from RA patients (*n* = 41; active, *n* =21; inactive, *n* =20) and HCs (*n* = 16) were detected by RT-PCR. **G**., **H**., **I**. The correlation between serum IL-33, ST2 and IL-10 in active RA patients. Statistical significance was evaluated by the Spearman’s rank correlation test. Data are means ± SEM. *p < 0.05, **p < 0.01, ***p < 0.001. ns = not significant.

To extend the knowledge about the previously reported that upregulated IL-33 and ST2 expression and increased IL-10 production could be detected in patients with RA and involved in the pathogenesis of RA [8, 14], we initially wanted to investigate the possible correlation between IL-33/ST2 and IL-10 in RA. In this study, 41 RA patients and 16 age- and gender matched healthy controls (HCs) were recruited (Table 1). As expected, we revealed that the serum and mRNA transcripts of IL-33 (Figure 1A, 1D), ST2 (Figure 1B, 1E) and IL-10 (Figure 1C, 1F) were dramatically upregulated in the patients with RA. Interestingly, active RA patients exhibited higher levels of IL-33 protein (Figure 1A) and mRNA (Figure 1D) compared with patients with inactive RA and HCs, but there were no distinct difference in ST2 (Figure 1B, 1E) and IL-10 (Figure 1C, 1F) protein and mRNA levels between active RA and inactive RA. We further analyzed serum levels of IL-33 with the aim of determining its possible correlation with ST2 and IL-10 expression in patients with RA. We found that serum IL-33 levels in active RA patients were positively correlated with IL-10 expression (Figure 1G), but no significant correlation was determined in all RA patients (data not shown). Furthermore, there was no correlation between serum ST2 and IL-10 in active RA patients (Figure 1H). Likewise, no significant correlation was detected between serum IL-33 and ST2 in active RA patients (Figure 1I). These data suggest that IL-33 expression is linked to anti-inflammatory action of IL-10 during disease progression of RA.

**Table 1:** Demographic and laboratory characteristics of the RA patients and healthy controls

Characteristics	RA (n=41)	HCs (n=16)
Age in years (mean)	50.7±12.78	50.3±8.87
Sex, no. Male/no. Female	9/32	4/12
Disease duration (years)	5.0 ± 4.76	-
DAS28 (mean ± SD)	3.8 ± 1.37	-
ESR (mm/h) (mean±SD) (%)	22.4 ± 18.67 (52.5%)	-
CRP (mg/L) (mean±SD) (%)	8.3 ± 11.37 (43.9%)	-
RF (IU/mL) (mean±SD) (%)	112.4 ± 193.20 (95.2%)	-
PSL drug responders, n (%)	30 (73.2%)	

Abbreviations: RA, Rheumatoid arthritis; HCs, Healthy controls; ESR, erythrocyte sedimentation rate; RF, rheumatoid factor; CRP, C-reactive protein; anti-CCP, anti-cyclic citrullinated peptide antibodies; DAS28, 28-joint Disease Activity Score; PSL, prednisolone.

### IL-10 deficiency accelerates arthritis by triggering IL-33/ST2 signaling

**Figure 2:**
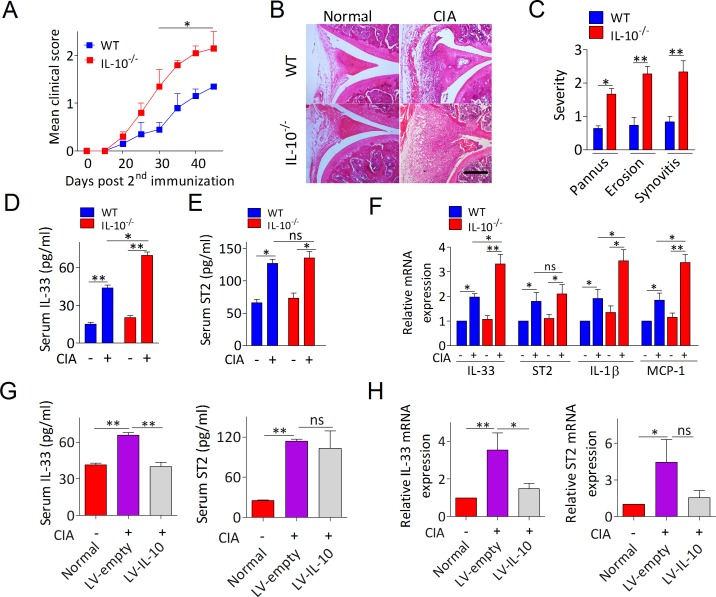
IL-10 ameliorates arthritis in CIA mice by suppressing levels of IL-33 **A**. Time-dependent changes in paw clinical arthritis severity scores of IL-10^−/−^ and WT mice with CIA after the second immunization. **B**. Representative H&E staining of knee joint sections of IL-10^−/−^ and WT mice with or without CIA. Scale bars, 100 μm. **C**. Histopathological score of synovitis, pannus, and erosion of bone and cartilage in the knee joint sections of IL-10^−/−^ and WT mice with CIA. **D**., **E**. The levels of serum IL-33 D. and ST2 E. levels were measured by ELISA (*n* = 5). **F**. The mRNA expressions of IL-33, ST2, IL-1β and MCP-1 in PBMCs from IL-10^−/−^ and WT mice with or without CIA were assessed by RT-PCR (*n* = 4). **G**., **H**. The levels of IL-33 and ST2 in serum G. and PBMCs H. from IL-10^−/−^ arthritic mice treated with LV-IL-10 or LV-empty were detected by ELISA and RT-PCR, respectively (*n* = 4-5). Normal, healthy IL-10^−/−^ mice without CIA; LV-IL-10, the knee joints of IL-10^−/−^ CIA mice injected with lentivirus-IL-10; LV-empty, the knee joints of IL-10^−/−^ CIA mice injected with lentivirus-control. Data are means ± SEM. *p < 0.05, **p < 0.01. ns = not significant.

To evaluate whether IL-10 is implicated in the blockade of IL-33/ST2 signaling during RA, we first investigated the development of collagen induced arthritis (CIA) in IL-10^−/−^ mice. We found that IL-10^−/−^ mice displayed more severe CIA than that in the WT mice (Figure 2A). Identification using histological analysis of knee joints suggests that more pronounced pannus formation, synovial hyperplasia, cartilage damage, and bone erosion in IL-10^−/−^ arthritic mice compared with WT arthritic mice (Figure 2B, 2C). By analyzing IL-33 and ST2 expression in serum and PBMCs from IL-10^−/−^ and WT CIA mice we observed the same behavior with RA patient’s cases (Figure 2D, 2E, 2F). Intriguingly, IL-10 deficiency substantially enhanced the expression of IL-33 in CIA mice, whereas ST2 level had no obviously change (Figure 2D, 2E, 2F). IL-10^−/−^ CIA mice also showed significantly upregulation of IL-1β and MCP-1, which are known to be regulated by IL-33/ST2 axis (Figure 2F). In CIA mice lacking IL-10 and administered IL-10 expressing lentivirus (LV-IL-10) directly into knee joint, ELISA and RT-PCR revealed a dramatically reduction of IL-33 rather than ST2 in serum (Figure 2G) and PBMCs (Figure 2H). Taken together, these results suggest that the anti-arthritic effect of IL-10 in CIA is likely involved in governing IL-33 pathway dependent inflammatory responses.

### IL-10 deficiency derives IL-33 production in inflammatory joints through decreasing STAT3 activity

**Figure 3:**
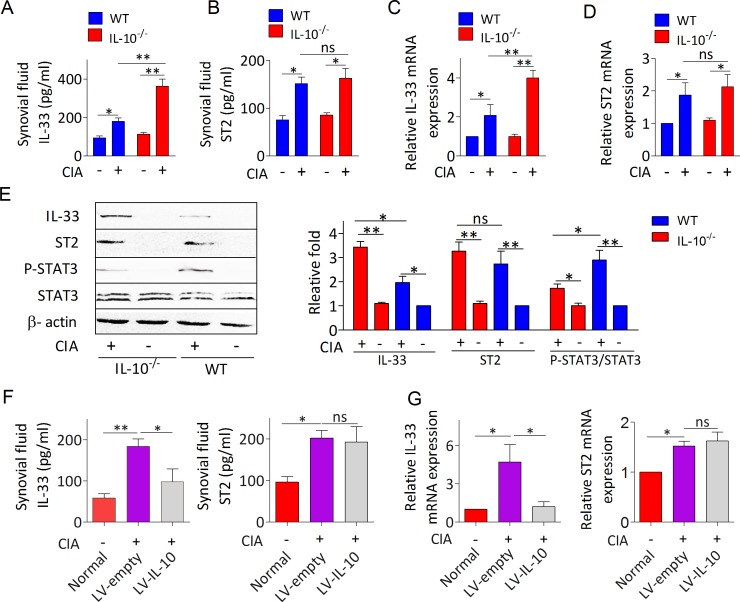
IL-10 restrains IL-33 expression in the inflammatory joint of CIA mice by activating STAT3 phosphorylation **A**., **B**. The levels of IL-33 A. and ST2 B. in synovial fluid of IL-10^−/−^ and WT mice with or without CIA were determined by ELISA (*n* = 5). **C**., **D**. RT-PCR analysis of IL-33 and ST2 mRNA expression in the synovial tissues of IL-10^−/−^ and WT mice with or without CIA. Data represent means ± SEM (*n* = 4-5). **E**. Western blot analyses and quantification of IL-33, ST2 and phosphorylated STAT3 in synovial tissues of IL-10^−/−^ and WT mice with or without CIA. The intensities of bands quantified densitometrically relative to the WT are represented as the bar graph. **F**., **G**. The levels of IL-33 and ST2 in synovial fluid F. and synovial tissues G. from IL-10^−/−^ arthritic mice treated with LV-IL-10 or LV-empty were detected by ELISA and RT-PCR, respectively (*n* = 4-5). Normal, healthy IL-10^−/−^ mice without CIA; LV-IL-10, the knee joints of IL-10^−/−^ CIA mice injected with lentivirus-IL-10; LV-empty, the knee joints of IL-10^−/−^ CIA mice injected with lentivirus-control. Data are means ± SEM.*p < 0.05, **p < 0.01. ns = not significant.

We also investigated whether IL-10 is responsible for limiting IL-33/ST2 elicited joint inflammation in CIA. Compared to mice without CIA, both IL-33 (Figure 3A, 3C) and ST2 (Figure 3B, 3D) levels were increased in synovial fluids and synovial tissues from mice with CIA. While IL-10^−/−^ arthritic mice exhibited higher levels of IL-33 compared with WT arthritic mice (Figure 3A, 3C), but ST2 expression was not affected (Figure 3B, 3D). Western blot analysis further demonstrated that synovial tissues of IL-10^−/−^ mice with CIA has higher expression of IL-33 proteins than those of WT mice with CIA (Figure 3E). Upregulated IL-33 expression in the synovium of IL-10^−/−^ mice with CIA was attributed to decreased STAT3 activity, evidenced by a 1.7 fold reduction of phosphorylated STAT3 level (Figure 3E). However, the attenuated expression of STAT3 in IL-10^−/−^ arthritic mice did not lead to obvious upregulation of ST2 (Figure 3E). Importantly, IL-33 but not ST2 expression was remarkably reduced in the synovial fluid (Figure 3F) and synovial tissues (Figure 3G) of IL-10^−/−^ arthritic mice intra-articularly injected with LV-IL-10 than in mice injected with LV-empty. These results suggest that IL-10 deficiency enhances IL-33 induced inflammatory response dependent on attenuation of STAT3 signaling.

### IL-33 level is elevated in macrophages of IL-10 deficient arthritic mice

**Figure 4:**
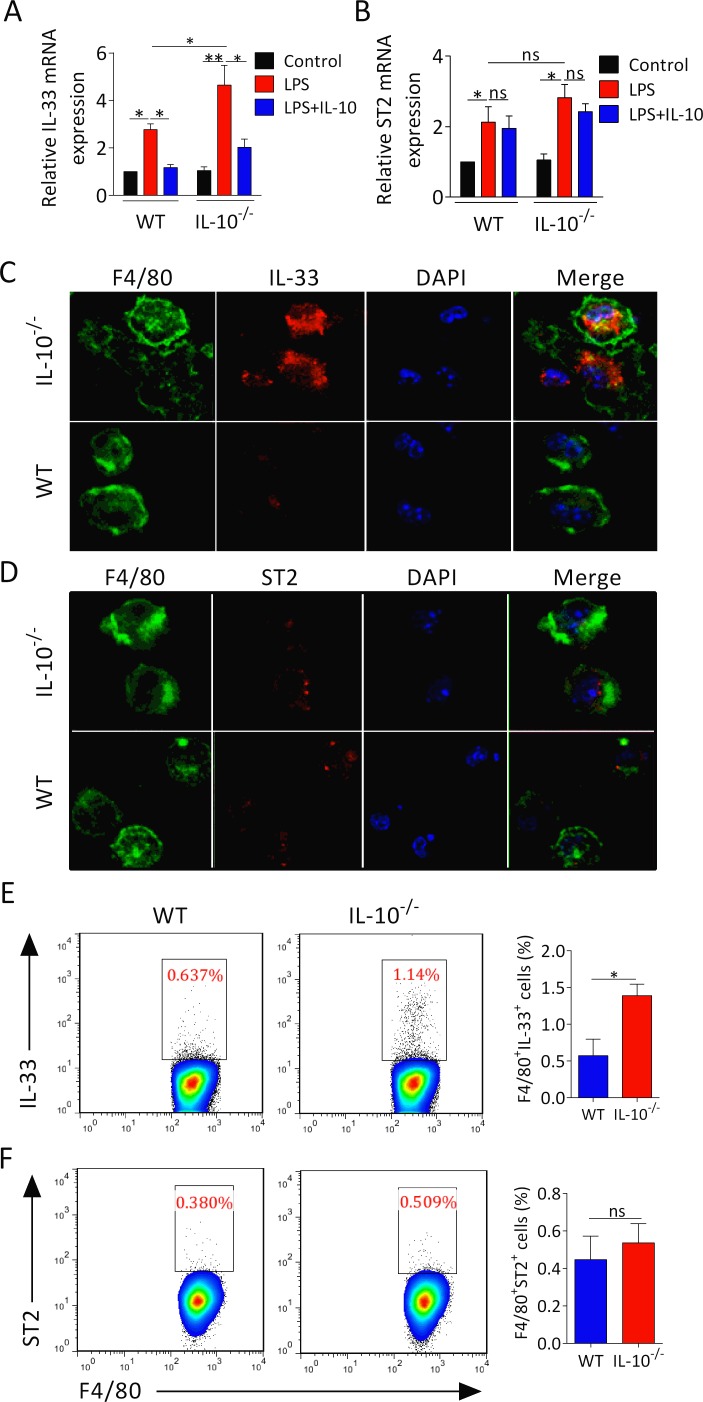
IL-10 deficiency increases IL-33 expression in macrophages **A**., **B**. Peritoneal macrophages from IL-10^−/−^ and WT mice were stimulated with LPS (1 μg/ml) for 1 hour, followed by treatment of 100 ng/ml IL-10 for 6 hours. RNA was isolated and the expression of IL-33 and ST2 was determined by RT-PCR. **C**., **D**. Confocal microscopy analyses IL-33 (red) and ST2 (red) expression in macrophages (green) from IL-10^−/−^ and WT mice with CIA. Bar = 25 μm. **E**., **F**. The cell number of IL-33^+^F4/80^+^ and ST2^+^F4/80^+^ macrophages from IL-10^−/−^ and WT mice with CIA was measured by flow cytometry. Data are means ± SEM. *p < 0.05, **p < 0.01. ns = not significant.

To determine whether the inhibitory function of IL-10 on macrophages downregulates IL-33 expression, peritoneal macrophages from IL-10^−/−^ and WT mice were treated with or without LPS in the presence or absence of IL-10. We found that the mRNA expression of IL-33 and ST2 was increased in both IL-10^−/−^ and WT macrophages upon LPS stimulation (Figure 4A, 4B). Moreover, IL-10^−/−^ macrophages treated with LPS displayed higher levels of IL-33 mRNA but not ST2 mRNA. (Figure 4A). Treatment of IL-10 dramatically reduced LPS induced IL-33 rather than ST2 expression in IL-10^−/−^ and WT macrophages (Figure 4A, 4B). Actually, confocal microscopy analyzing also confirmed the importance of IL-10 in restricting IL-33 expression in macrophages. We found that IL-10^−/−^ arthritic mice had substantially increased IL-33 expression in F4/80^+^ phenotype macrophages (Figure 4C), whereas the expression of ST2 on F4/80^+^ macrophages was not altered (Figure 4D). Moreover, the proportion and the number of IL-33^+^F4/80^+^ macrophages but not ST2^+^F4/80^+^ macrophages were elevated in IL-10^−/−^ arthritic mice (Figure 4E, 4F). These results suggest that IL-10 is crucial for restraining IL-33 expression in macrophages.

### IL-10 abrogates IL-33 induced NF-κB activation

**Figure 5:**
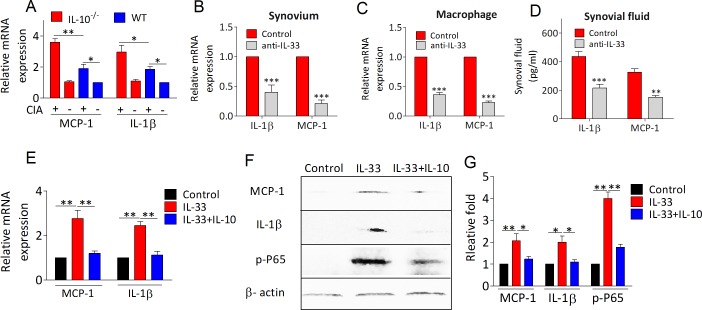
IL-10 suppresses IL-33/ST2 pathway induced proinflammatory factors production **A**. The mRNA levels of MCP-1 and IL-1β in synovium from IL-10^−/−^ and WT mice with or without CIA were detected by RT-PCR. **B**., **C**., **D**. IL-10^−/−^ mice were immunized with CII/CFA on day 0 and boosted with CII/CFA on day 21. IL-10^−/−^ CIA mice were intraperitoneally injected with anti-IL-33 antibodies (100 ug per mouse) every 4 days. The mice were sacrificed on day 41. The levels of MCP-1 and IL-1β were measured by means of RT-PCR or ELISA in synovium B., macrophage C., and synovial fluid. D. **E**. Peritoneal macrophages from WT mice were stimulated with IL-33 (100 ng/ml) alone or IL-33 (100 ng/ml) plus IL-10 (100 ng/ml) for 4 hours. RNA was isolated and the expression of IL-33 and ST2 was determined by RT-PCR. **F**., **G**. Representative results and statistical analysis of MCP-1, IL-1β and NF-κB (P65) by western blot assay under IL-33 and/or IL-10 stimulation. Data are means ± SEM. *p < 0.05, **p < 0.01, ***p < 0.001.

We next examined whether the anti-arthritic role of IL-10 is involved in dampening IL-33/ST2 signaling enhanced proinflammatory milieu. As shown in Figure 5A, proinflammatory factors IL-1β and MCP-1 mRNA expression were significantly elevated in synovial tissues of IL-10^−/−^ arthritic mice. Interesting, IL-33 blockade with anti-IL-33 antibody in IL-10^−/−^ arthritic mice markedly attenuated the expression of IL-1β and MCP-1 in synovium, macrophages and synovial fluid of knee joint, suggesting IL-10 deficiency increased inflammation depending on IL-33/ST2 activation (Figure 5B, 5C, 5D). Furthermore, IL-33 induced IL-1β and MCP-1 mRNA expression was fully abolished in peritoneal macrophages upon recombinant IL-10 treatment (Figure 5E). In support of this, both IL-1β and MCP-1 protein levels were dramatically decreased in macrophages treated with IL-33 in the presence of IL-10 by western blot analysis (Figure 5F, 5G). To analyze the mechanism abrogated by IL-10 in IL-33 enhanced inflammatory responses, we monitored IL-33 induced activation of NF-κB and p38 signaling pathway in macrophages. We found that phosphorylation of NF-κB p65 (Figure 5F, 5G) and p38 (Supplemental Figure 1) could be significantly increased by IL-33 stimulation, whereas IL-33 induced NF-κB activation could also be blocked by IL-10 (Figure 5F, 5G). Interestingly, IL-10 did not suppress IL-33 induced p38 activation and downstream IL-13 expression (Supplemental Figure 1). These results indicate that the immunosuppressive effects of IL-10 might be achieved by restraining IL-33 activated NF-κB signaling pathway.

## DISCUSSION

The present study supports the importance of IL-33/ST2 axis in RA and reveals a novel mechanism by which IL-10 signaling ameliorates autoimmune arthritis through inhibition of IL-33 expression in macrophages and blockade of proinflammatory cytokines and chemokines production in response to IL-33. A major pathway by which IL-10 protects against arthritic inflammation is likely to be via selectively impairing IL-33 activated NF-κB signaling rather than p38MAPK signaling activation in macrophages.

Despite preliminary studies have pointed to a contribution of IL-33/ST2 signaling in releasing inflammation in murine models [9, 15, 16], the detailed analysis of IL-33 and ST2 expression in different disease stages of RA patients has not yet been elucidated. The findings of this study revealed a strong positive correlation between IL-33 and IL-10 in serum and PBMCs from patients with active RA, indicating that an intrinsic mechanistic interaction between proinflammatory cytokine and antiinflammatory cytokine. Many studies have indicated that IL-33 level will reach its peak in the early phase of the inflammatory process of RA, whereas anti-inflammatory cytokines expression predominates later in the disease process [15, 17]. Indeed, upregulated IL-10 expression has been identified in many human autoimmune diseases and in animal models [14, 18, 19]. It is reasonable to speculate that IL-33 elicited inflammatory response is able to provide a feedback loop for IL-10 upregulation, which plays an anti-inflammatory role in RA.

It is widely recognized that IL-33/ST2 signaling is essential for development of autoimmune inflammation, including RA [3, 16]. The blockade of IL-33/ST2 system is likely to give potential opportunities for the treatment of RA disorder. In this study, we surprisingly found that loss of IL-10 enhanced IL-33 expression in the local joint by downregulating STAT3 activation during CIA. Conversely, overexpression of IL-10 in the joints of IL-10 deficient arthritic mice suppressed IL-33 expression and IL-33 derived inflammation. However, ST2 expression still constant in all samples. Within our knowledge, ST2 can exist in two different splice variants leading to the synthesis of transmembrance receptor (ST2L) and soluble molecule (sST2) [7]. sST2 acts as a decoy receptor that prevents the interaction of ST2L with IL-33, whereas ST2L binding its ligand IL-33 activates inflammatory responses in autoimmunity [7]. Although the level of sST2 is elevated in the serum and synovial fluid of RA, ST2L expression is dependent on pro- or anti- inflammatory surroundings. For instance, ST2L expression in Th2 cells is enhanced by IL-4 and suppressed by IFN-γ [16]. This observation suggests that the isoforms of ST2 may lead to differential respond to IL-10. Furthermore, the inflammatory state is also involved in the inhibitory function of IL-10 in ST2 expression during RA. Additional work is required to determine the role of IL-10 in the expression of isoforms of ST2 in the disease stages of RA.

It has been reported that endothelial cells and fibroblasts are a key source of IL-33 in inflamed synovium, but the fibroblasts seem to express only low levels of IL-33 [15, 20]. More recently, evidence has shown that activated macrophages and synoviocytes appear to be the main producers of IL-33 in an autocrine loop [21]. In this study, we identified that IL-10-STAT3 pathway for restricting IL-33 seems to be macrophages-specific. In the mouse arthritic models, IL-10 deficiency not only substantially up-regulated IL-33 expression in F4/80^+^ macrophages but also elevated the frequency of IL-33^+^F4/80^+^ macrophages. Unlike its function in suppressing IL-33 expressing macrophages, IL-10 did not inhibit ST2 expressing in macrophages. The reason for this difference may be because inflammation microenvironment and cell type lead to ST2 lack of effective responsive to IL-10 during CIA.

**Figure 6:**
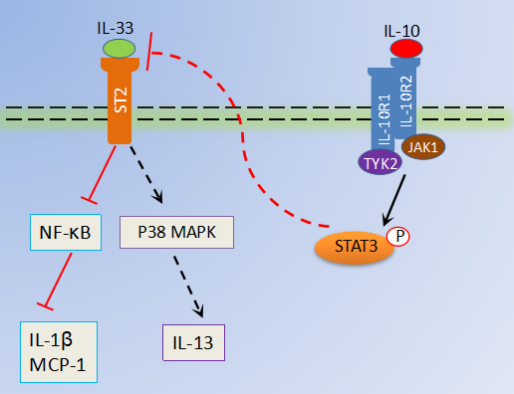
Schematic representation of antiinflammatory role of IL-10 in RA IL-10, binds its receptor IL-10R, which suppresses IL-33 production in macrophages via activation of STAT3 phosphorylation. Moreover, inflammatory factors (MCP-1 and IL-1β) secretions by IL-33/ST2 activated NF-κB in macrophages are also abrogated by IL-10 signaling, which further restrains excessive joint inflammation in RA. However, IL-33/ST2 induced p38MAPK signaling pathways in macrophages are not restrained by IL-10.

Previous studies have identified that IL-33 might acts via autocrine and paracrine fashion in immune responses [9, 16, 20], whereas the capability of IL-33 in RA has not been fully elucidated. Our data showed that depletion of IL-33 effectively decreased inflammatory mediators IL-1β and MCP-1 production in macrophages and local joint of IL-10 deficient CIA mice. Moreover, IL-33 induced IL-1β and MCP-1 expression in macrophages was suppressed by recombinant IL-10. It has been demonstrated that IL-33 potently induced MCP-1 and IL-1 production which in turn are able to enhance IL-33 expression in synoviocytes [9, 21]. Therefore, our finding reported here that IL-10 signaling not only blocked IL-33 production but also restricted IL-33 induced cascade of cytokine/chemokine events in macrophages during RA. The activity of IL-33 as a signaling molecule is known to be mediated by multiple pathways, including NF-κB and MAPKs [22]. As reported before, both NF-κB and P38 phosphorylation were strongly elevated in macrophages in the presence of IL-33. Strikingly, IL-10 significantly impaired IL-33 activated NF-κB signaling but failed to inhibit IL-33 activated p38 signaling. It seems that IL-33 activated NF-KB signaling pathway mainly responsible for the production of proinflammatory factors such as IL-1β, MCP-1, IL-6 and TNF. However, the production of Th2 cytokines by immune cells in response to IL-33 is dependent of MAPK signaling pathway [16]. It is likely that IL-33 activated signaling pathway depending on the cytokine milieu and different cell types. Thus, it is reasonable that IL-10 contribute to the inhibition of IL-33 amplified inflammation through selectively restrains NF-κB activation in macrophages rather than p38 signaling pathway (Figure 6). Nevertheless, how IL-10 suppresses IL-33 induced NF-κB signaling pathway are issues that require further study.

In summary, these findings may improve our understanding of the molecular mechanisms involved in anti-arthritic effect of IL-10 and identify that IL-10 protects against RA via inhibiting IL-33/ST2 signaling amplified inflammatory responses. We also show that IL-33/ST2 signaling in macrophages is an important direct target of IL-10. STAT3 and NF-κB signaling are essential for the protective effect of IL-10 against IL-33 induced inflammatory responses cascade. These findings may provide new insights into future therapeutic targets for RA.

## MATERIALS AND METHODS

### Patients and HCs

Forty-one patients with RA (mean age±SD: 50.7±12.78 years; male/female: 9/32) and sixteen sex and age-matched healthy controls (HCs) (mean age±SD: 50.3±8.87 years; male/female: 4/12) in this study were recruited from Peking University Shenzhen Hospital (Table 1). The classification of RA met the American College of Rheumatology criteria [23]. Based on 28-joint disease activity score (DSA28) [24], RA patients were divided into two groups, active (DAS28 ≥ 3.2) and inactive (DAS28 < 3.2). For cytokine measurement, peripheral blood samples from RA patients and sex and age-matched HCs were collected (Table 1), and then they were isolated into serum and PBMCs according to our previous protocol [19]. Serum and PBMCs samples were stored at -80°C until cytokines were determined. All patients provided informed consent, and study approval was obtained from our local ethics committee.

### Mice

Ten to 14-week-old male C57BL/6J wild-type (WT) mice, C57BL/6J derived IL-10 knockout (IL-10^−/−^) mice and DBA/1J mice were purchased from The Jackson Laboratory (Bar Harbor, ME, USA) and maintained under specific pathogen-free conditions in the laboratory animal center of Shenzhen University (Shenzhen, China). We also generated IL-10 deficient mice with DBA/1J background by backcrossing the original IL-10^−/−^ C57BL/6J with DBA/1J mice. The mice were typed by PCR and IL-10^−/−^ DBA were further backcrossed into DBA/1J. Mice (IL-10^−/−^ DBA/1J, age 8-12wk) from the fifth generation were used for the IL-10 treatment and IL-33 neutralization experiments. All animal experiments were approved by the Institutional Animal Care and Use Committee of Shenzhen University.

### CIA induction

Induction of CIA was performed as previously described [12]. Briefly, chicken CII (Chondrex) emulsified in CFA containing M. tuberculosis (Chondrex). On day 0, 200 μl emulsions were injected intradermal at the base of the mouse tail. After two weeks, a booster injection was administered neat the primary injection site. Mice were monitored for signs of arthritis every day from the day of the booster injection until to the day of 45. On day 30 after boost injection, samples from CIA mice were harvested for performing experimental analysis in this study.

### Administration of lentivirus-IL-10 and anti-IL-33 in CIA

IL-10^−/−^ DBA/1J and Wild type mice were inoculated intradermally on day 0 and 21 with 200 μl bovine type II collagen (CII; Chondrex) emulsified in CFA containing *Mycobacterium tuberculosis* (Chondrex). For the lentivirus-IL-10 treatment, A total of 10^7^ TU/ml lentivirus containing the mouse IL-10 gene (LV-IL-10) or empty lentivirus control (LV-empty) (GeneChem, Shanghai, China) was injected intra-articularly into the knee joints on day 5 and 18 after the first CII immunization. For details of the lentivirus see http://www.genechem.com.cn. Samples were harvested from LV-IL-10 and LV-empty treated CIA mice on day 35 after the first immunization. For neutralizing IL-33 experiments, 100 μg anti-IL-33 antibodies (R&D Systems, USA) were given intraperitoneally on day 14 and followed with same dose every four days apart. Mice were sacrificed on day 41, and samples were isolated for performing experimental analysis.

### Clinical and histological assessment of arthritis

Arthritis severity was assessed by mean clinical scores as follows: grade 0 = no swelling; grade 1 = slight swelling and erythema; grade 2 = pronounced swelling; and grade 3 = joint rigidity. For histological assessment, knee joint sections were stained with H&E. Histopathologic scoring of joint damage was performed under blinded conditions for evaluating synovitis, cartilage degradation, and bone erosion [12].

### Macrophages and synovial fluid preparation

Peritoneal and joint macrophages and synovial fluids were harvested based on a previously described protocol [12]. Macrophages were used for treatment or further experimental analysis. Synovial fluid samples were stored at -80°C prior to performing ELISA assay.

### ELISA

The levels of human and mouse cytokines were quantified using ELISA kits, according to the manufacturer’s instructions.

### Confocal microscopy

Macrophages were fixed in with 4% paraformaldehyde in PBS, permeabilized with 0.1 % Triton X-100 for 5 min and blocked with 3% BSA for 1 h. After being washed three times with PBS, cells were incubated with FITC-anti-F4/80 antibody (eBioscience, San Diego, CA, USA), PE-anti-IL-33 antibody (R&D systems, Minneapolis, MN, USA) and APC-anti-ST2 antibody (R&D systems, Minneapolis, MN, USA) in 3% BSA at 37°C for 1 h. The cells were washed in PBS, and nuclei were stained with DAPI. Subsequently, the cells were detected with a Leica TCS SP5 confocal laser-scanning fluorescence microscope (Leica Microsystems, Buffalo Grove, IL, USA).

### Quantitative real-time PCR (RT-PCR)

RNA was extracted by TRIzol reagent (Invitrogen). cDNA was prepared using the iScript cDNA Synthesis Kit (Bio-Rad), according to the manufacturer’s instructions. Primer sequences are detailed in Table 2. RT- PCR amplification reaction was prepared with the SYBR Green PCR Kit (Bio-Rad) and performed using the ABI 7500 Fast Real-Time PCR System (Applied Biosystems). Relative target gene expression was calculated by normalization to β-actin or GADPH value using the 2^−ΔΔct^ method.

**Table 2:** primer sequences

	Gene	Sense primer	Antisense primer
Human	IL-10	TCCTCCAGCAAGGACTCCTTT	CTGCCTAACATGCTTCGAGATC
	IL-33	ATGAAGCCTAAAATGAAGTATTCA	CTAAGTTTCAGAGAGCTTAAACAAG
	ST-2	CTTGATTGATAAACAGAATG	CTGATCCAGATACTGTTGAA
	actin-β	CCTGACTGACTACCTCATGAAG	CGTAGCACAGCTTCTCCTTA
Mouse	IL-33	ATTTCCCCGGCAAAGTTCAG	AACGGAGTCTCATGCAGTAGA
	ST2	TGTATTTGACAGTTACGGAGGGC	ACTTCAGACGATCTCTTGAGACA
	IL-1β	CCTTCCAGGATGAGGACATGA	TGAGTCACAGAGGATGGGCTC
	MCP-1	TAAAAACCTGGATCGGAACCAAA	GCATTAGCTTCAGATTTACGGGT
	GAPDH	ACCACAGTCCATGCCATCAC	TCCACCACCCTGTTGCTGTA

### Western blot analysis

For Western blotting, the lysates from tissue and treated cells were prepared, then separated by SDS-PAGE, and transferred to PVDF membranes. The following primary Abs used for western blot analysis: anti-IL-33 (Abcam, Cambridge, UK), anti-ST2 (Abcam, Cambridge, UK), anti-IL-1β (Cell Signaling Technology, Danvers, MA, USA), anti-MCP-1 (Santa Cruz Biotechnology, California, USA), anti-STAT3 (Cell Signaling Technology, Danvers, MA, USA), anti-pSTAT3 (Cell Signaling Technology, Danvers, MA, USA), anti-NF-κBp65 (eBioscience) and anti-β-actin (Cell Signaling Technology, Danvers, MA, USA). Immune reaction bands were detected by using horseradish peroxidase-labeled, species-specific secondary antibodies (Cell Signaling Technology) and enhanced chemiluminescence analysis (EMD Millipore, Billerica, MA, USA), and viewed by Kodak Image Station 4000MM (Eastman Kodak, Rochester, NY, USA).

### Flow cytometry assay

Cell-surface and intracellular staining was done as reported previously [12]. Cell-surface staining was carried out using FITC-anti-F4/80 (eBioscience, San Diego, CA, USA) and APC-anti-ST2 (R&D systems, Minneapolis, MN, USA). For intracellular staining of PE-anti-IL-33 (R&D systems, Minneapolis, MN, USA), cell stimulation cocktail was added and cultured for the last 5hs before flow cytometric analysis.

### Statistical analysis

Data were expressed as the mean±SEM of at least three independent experiments. Statistical analysis was performed using Graphpad version 5.0 software (GraphPad Software, La Jolla, CA, USA). A two-tailed Student’s t-test was used for two group statistical comparison. Where appropriate, the Bonferroni posttest and a Mann-Whitney *U* test were used to analyze multiple comparisons and non-parametric data, and correlations were examined by Spearman’s rank correlation test. Differences were considered statistically significant with *p* < 0.05.

## SUPPLEMENTARY MATERIALS FIGURES


