# Supplementary material for: IL-33/ST2-mediated inflammation in macrophages is directly abrogated by IL-10 during rheumatoid arthritis
Source: Oncotarget. 2017 Mar 16;8(20):32407–18. doi: 10.18632/oncotarget.16299 (PMC5464798; doi:10.18632/oncotarget.16299)
Supplement: Supplementary file 1 [file oncotarget-08-32407-s001.pdf]

IL-33/ST2-mediated inflammation in macrophages is directly abrogated by IL-10 during rheumatoid arthritis

Supplementary Material

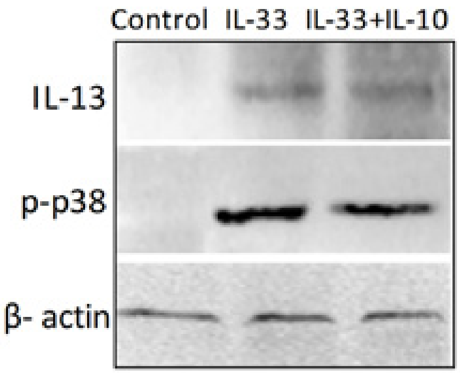

Supplemental Figure 1: IL-10 does not affect IL-33 modulated p38 signaling pathway. Peritoneal macrophages from WT mice were treated with IL-33 (100 ng/ml) or IL-33 (100 ng/ml) plus IL-10 (100 ng/ml) for 15 min or 4 hours. p38 phosphorylation and IL-13 expression was measured by western blot respectively.
